# Skin Basement Membrane: The Foundation of Epidermal Integrity—BM Functions and Diverse Roles of Bridging Molecules Nidogen and Perlecan

**DOI:** 10.1155/2013/179784

**Published:** 2013-03-21

**Authors:** Dirk Breitkreutz, Isabell Koxholt, Kathrin Thiemann, Roswitha Nischt

**Affiliations:** ^1^Department of Dermatology, University of Cologne, Kerpener Strasse 62, 50937 Cologne, Germany; ^2^DGZ, Post Office Box, German Cancer Research Center (DKFZ), Im Neuenheimer Feld 280, 69009 Heidelberg, Germany

## Abstract

The epidermis functions in skin as first defense line or barrier against environmental impacts, resting on extracellular matrix (ECM) of the dermis underneath. Both compartments are connected by the basement membrane (BM), composed of a set of distinct glycoproteins and proteoglycans. Herein we are reviewing molecular aspects of BM structure, composition, and function regarding not only (i) the dermoepidermal interface but also (ii) the resident microvasculature, primarily focusing on the *per se* nonscaffold forming components perlecan and nidogen-1 and nidogen-2. Depletion or functional deficiencies of any BM component are lethal at some stage of development or around birth, though BM defects vary between organs and tissues. Lethality problems were overcome by developmental stage- and skin-specific gene targeting or by cell grafting and organotypic (3D) cocultures of normal or defective cells, which allows recapitulating BM formation *de novo*. Thus, evidence is accumulating that BM assembly and turnover rely on mechanical properties and composition of the adjacent ECM and the dynamics of molecular assembly, including further “minor” local components, nidogens largely functioning as catalysts or molecular adaptors and perlecan as bridging stabilizer. Collectively, orchestration of BM assembly, remodeling, and the role of individual players herein are determined by the developmental, tissue-specific, or functional context.

## 1. Introduction 

In skin the epidermis represents the outer barrier of the organism, providing protection against physical, chemical, and microbial impacts of the environment. It should be mentioned beforehand that skin in general is serving multiple other functions (e.g., sensing touch, pain, temperature, and priming immune responses), thus representing our second largest organ only surpassed by the vascular system. However, these other issues are beyond the scope of this paper. The skin consists of two morphologically distinguishable compartments, the epidermis and the dermis, which communicate in various ways and at different levels to establish, maintain, or restore tissue homeostasis. While in skin the dermis bears the main mechanical load and provides also insulation, the vital barrier function at the outer surface is accomplished by the epidermis which is a constantly renewing, stratifying, and keratinizing epithelium [1, 2]. Special lipids and tight junctions between epidermal cells (keratinocytes) in upper layers prevent penetration or loss of water [3, 4], and finally the formation of cornified envelopes, an alloy of highly cross-linked proteins and proteolipids, is warranting chemical resistance [5–9]. Mechanical resistance of the epidermis relies on the intracellular keratin filaments which form via epithelia-specific junctions, the desmosomes a large, continuous intraepithelial network ([2, 4, 10] detailed reviews). The dermal tensile strength and elasticity are defined by its extracellular matrix (ECM) properties with type I and III collagen fibrils, microfibrils, and elastic fibers, embedded in a ground substance of proteoglycans [11]. The boundary between the two skin compartments provides the basal lamina or basement membrane (BM), a highly specialized ECM structure, which physically separates the two compartments rendering primarily a stabilizing, though still dynamic interface and a diffusion barrier [12–19]. 

Besides their prominence in skin, BMs support all epithelia and endothelia, enwrap Schwann cells and nerve extensions [20, 21], muscles [22, 23], tissue compartments like fat, and whole organs [14]. With highly specialized modifications BMs are essential for function in the glomeruli of the kidney [24–26], in nerve synapses [27], and neuromuscular junctions [23, 28–30]. Apart from structural properties, the dermoepidermal BM has gate-keeping functions which control cell traffic and diffusion of bioactive molecules in both directions. In addition, the BM is binding a variety of cytokines and growth factors, serving a reservoir for their controlled release [31–34]. This plays a crucial role during physiological remodeling or repair processes after injury, while under pathologic conditions such as inflammation the release of factors is further enhanced due to vast BM destruction, being also part of the activating stroma reaction in cancer [35, 36]. Thus, the pivotal role of altered cell interactions with ECM becomes especially apparent in healing wounds or in invading tumors, where epithelial cells are confronted with other, newly accessible ECM molecules, their proteolytic fragments, or cleavage sites (neoepitopes) in the surrounding stroma [37–40]. Main cell surface mediators of those cell matrix interactions are *α*-/*β*-dystroglycan [41–44], syndecans [29, 45–47], and certain integrins being members of a large family of heterodimeric transmembrane proteins ([48–51]; for general review: [19]). Integrins are intracellularly associated via adapter proteins with actin microfilaments, which is crucial for both cell adhesion and migration, becoming particularly apparent in tumor invasion and metastasis ([52, 53]; reviewed by [54]), and ECM-mediated signalling [55–59]. The only exception is integrin *α*6*β*4 which is normally connected to the basal part of the keratin network via the long cytoplasmic tail of the *β*4 subunit and internal plaque proteins (outlined later). Thus, during BM assembly *α*6*β*4 becomes an integral part of hemidesmosomes which represent the firm epidermal adhesion points to the BM [60–62]. In the normal balanced state integrins show a polarized distribution, integrins *α*2*β*1 and *α*3*β*1 covering lateral and ventral surfaces of basal cells, while *α*6*β*4 is largely restricted to the ventral site opposed to ECM or BM [48, 49, 51]. Strikingly, in skin wounds or tumors the pericellular integrin distribution, including then also *α*6*β*4, largely expands into suprabasal layers, which reflects a severe reduction in cell and tissue polarity [63–65]. Last not least, particular properties or microheterogeneity of BMs is supposed to contribute to the niche of tissue-specific stem or progenitor cells [66–72].

## 2. Molecular Building Blocks of Basement Membranes 

**Figure 1:**
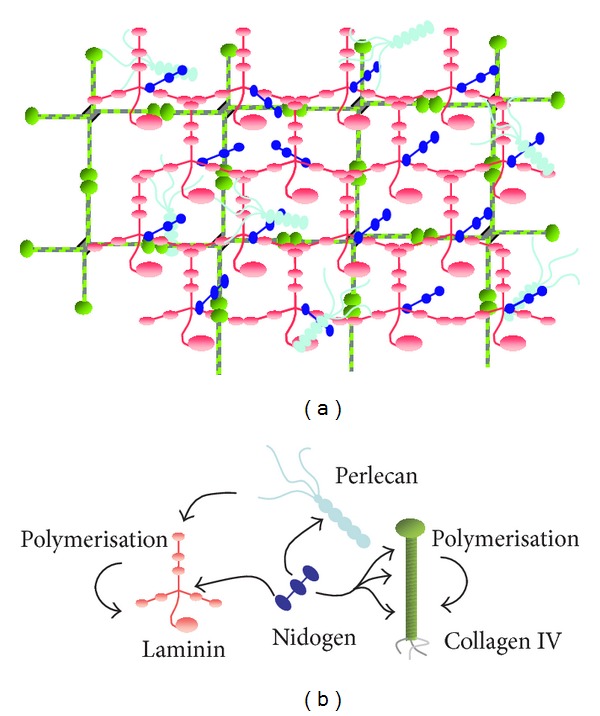
Schematic view of the basement membrane (BM). (a) The general molecular array leading to the mat-like BM texture and (b) the interactions between the four major individual BM components based on *in vitro* binding data.

**Figure 2:**
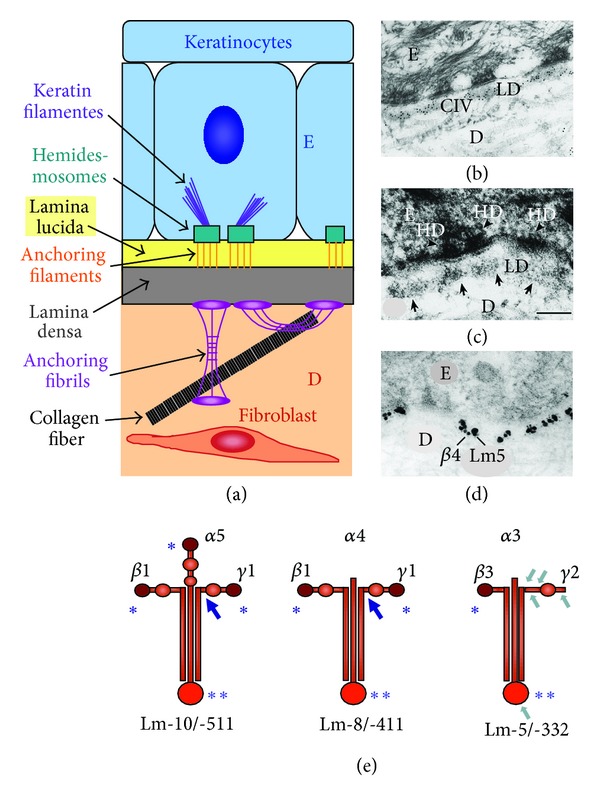
Ultrastructural elements (a–d) of the basement membrane (BM) zone in skin, ultrastructural alignments (b–d), and prototypes of laminin isoforms (e). The cartoon (a) depicts the anchoring structures between epidermis (E) and dermis (D) corresponding to the ultrastructure of the collagen-epidermal interface (b) of a 3D coculture of keratinocytes with fibroblasts, resembling skin. Immune-EM demonstrates the coalignment of collagen IV with the *lamina densa* (c) and colocalization of integrin *α*6*β*4 with laminin-332 ((d); small/large gold particles). Three laminin subtypes, being also present in adult skin, are shown in (e), represented by the main adult BM-type laminin-511, the vascular laminin-411, and laminin-332 found in anchoring filaments. Like laminin-511, most isoforms carry three N-terminal self-assembly sites (∗) required for two-dimensional polymerization. Some other like laminin-411 have only two, and, as an exception, laminin-332 has only one of those “sticky” sites. Common to all are the C-terminal cell-binding sites (∗∗); large arrows point to the *γ*1 nidogen-binding domain. Further typical for laminin-332 is extensive proteolytic processing with the major cleavage sites (marked by small arrows) at the short arm of the *γ*2 and the C-terminus of the *α*3 chain (see also: [78, 125]). (Slightly modified from [18] [with kind permission from Springer Science + Business Media]).

In general, BMs contain at least one member of the four protein families or subtypes of laminin, type IV collagen, nidogen, and perlecan, a heparan sulfate proteoglycan ([14, 73, 74]; for review: [19, 75, 76]) which determines their common structure. To some extent the tissue-specific functional diversity is accomplished by differential expression of respective isoforms [16, 31, 77–80]. As the principal structural elements, laminin and collagen IV form distinct networks [81] which become noncovalently interconnected by mono- or oligomeric nidogen [41, 82, 83] and perlecan, able to form irregular polymers [74, 84] (Figures 1(a) and 1(b)). Additional components may be involved as well such as fibrillin [85, 86], collagen V [87], perhaps also the BM-associated collagens XV and XVIII [31, 88] and extracellular matrix protein 1/ECM1 [89, 90]. Most of the laminin isoforms are able to self-assembly occurring via the N-terminal globular domains at the short arms of their *α*, *β*, and *γ* chains by noncovalent bonds, forming large two-dimensional sheets ([19, 78, 81, 91, 92], also for review) (Figures 1 and 2(e)). This reversible interaction allows local disassembly when needed, for example, during tissue remodeling. Crucial for cell or tissue fate and function is cell adhesion to laminins with the main cell binding site residing in the C-terminal globular domain on the long arm of the *α* chain [14, 20, 93, 94]. The collagen IV molecules, on the contrary, are covalently cross-linked by disulfide bridges via their noncollagenous C- and globular N-terminus, giving rise to a very stable “chicken-wire”-like meshwork of high chemical resistance [14, 84, 95]. Thus, the mechanical BM robustness is mostly determined by the collagen IV scaffold [96], whereas for the initial steps of BM assembly *in vivo* laminin is essential [14, 97, 98].

As mentioned above, both nidogen and perlecan, not forming structured polymers, are bridging these scaffolds by their multiple binding sites for laminin and collagen IV, including the perlecan heparan sulfate chains [84], as well as for each other ([19, 99–103], also for review). Complete perlecan deficiency is lethal for mouse embryos at the midgestational stage [104, 105], and the deletion of both nidogens is perinatally lethal [106]. 

The predominating nidogen-1 [82, 83, 107] and the later discovered nidogen-2 as second mammalian isoform [108–110] are ubiquitous BM proteins though nidogen-2 shows more restricted expression patterns throughout development and some tissue specificity in adulthood (comprehensive review, [111]). Both isoforms interact *in vitro* with many other BM molecules, in particular laminin and collagen IV, implying nidogens as essential integrating elements for BM assembly [99, 101, 109, 110]. Thus, primarily nidogen has been considered as the main cross-linker between laminins and collagen IV, revealing very high affinity to the laminin *γ*1 chain [101, 112, 113] but also the *γ*3 chain [111, 114, 115]. Contrarily, a major regulatory role has been assigned to perlecan [116] which implements a high negative charge in BMs through its three heparan sulfate side chains, providing a diffusion barrier as well as anchoring port [31]. However, a recent report has presented strong evidence that perlecan aggregates function as more stable connecting bridges [84] though the binding to laminin and collagen IV via the heparan sulfate chains seems to be of rather low specificity [19]. According to the BM ultrastructure (Figures 2(b) and 2(c)) observed by transmission electron microscopy (EM; standard fixation), the laminin/collagen IV polymers form the body of the *lamina densa* below the “empty” *lamina lucida*, which has been confirmed by immune EM [12, 75, 117, 118]. At this point it should be noted that the *lamina lucida* is not detectable in EM specimens fixed by cryopreservation, indicating that the *lamina lucida* reflects rather an artificial structure than real BM topography. Spacing and actual orientation of BM molecules could be determined by applying epitope-specific antibodies [12]. Anchorage of the epidermis to the BM (schematic view in Figure 2(a)) is accomplished by hemidesmosomes, consisting of the intracellular plaque proteins plectin and bullous pemphigoid antigen 1/BPAG1 [2, 119] which link the keratin filaments to the transmembrane proteins integrin *α*6*β*4 [60, 120], tetraspanin CD151 [121], and collagen XVII (BP180 and BPAG2; [122, 123]). Integrin *α*6*β*4, the only integrin associated with keratins, binds to laminin-332 (laminin-5; [79]) (colocalization shown in Figure 2(d)), which is not self-polymerizing and forms together with the extracellular domain of collagen XVII [124], the anchoring filaments spanning the whole BM. This requires specific molecular tailoring of laminin-332 by sequential proteolytic processing (Figure 2(e)) [65, 125–127]. Initially keratinocytes attach to unprocessed laminin-332 via integrin *α*3*β*1 (associated with the actin cytoskeleton), forming focal adhesion contacts, which promotes cell migration. Sequential cleavage of distinct laminin modules leads to strong cell adhesion via *α*6*β*4 and hemidesmosome formation (“laminin-5r”; [128, 129]). Contrarily, further truncation of laminin-332 during wound regeneration or tumor invasion promotes again cell motility [78, 125, 130–132]. Tetraspanin CD151 seems to mediate the transitions between these stable and dynamic cell-matrix contacts [119, 121, 133], which is also involved in tumor cell migration [134]. Several proteases seem to participate in these processes, for example, plasmin [50, 126], matrix metalloproteinases like MMP-2, -3, -14/MT1-MMP [135, 136] and other astacin family members like bone morphogenetic protein 1/BMP1, mammalian tolloid/mTLD, and mammalian tolloid-like metalloproteases/mTLL [137]. Some divergence between data may relate to the tissue type or state such as physiologic or pathologic turnover, activation by inflammatory reactions, or tumor invasion and metastasis, upregulating MMPs like MMP-14 [136] and in many tumors the surface-protease hepsin [132].

The BM is connected to the dermis underneath by anchoring fibrils, loop structures of collagen VII, which bind to laminin-332 by their NC-1 domains [138] and are interwoven with the fabric of collagen I and III fibrils [139–141]. Collectively these adhesion complexes are essential for the structural and functional integrity of skin [12]. Thus, inherited or acquired defects of those BM or BM-associated molecules result mostly in severe or lethal blistering diseases [139, 142, 143]. 

For the following it should be explicitly stated that in skin the components collagen IV and VII, and perlecan are synthesized by both epidermal keratinocytes and dermal fibroblasts, while the main source of nidogens are the fibroblasts [144–146] and of laminin-332 and -511 the keratinocytes [125, 144]. The first detectable isoform in the embryo laminin-111 is predominant in BMs during early development and is in most BMs like in skin successively replaced to a large extent by laminin-511. That is crucial for organogenesis and becomes the most abundant isoform in the adult organism [41, 147, 148]. Similarly laminin-211, a minor component in embryonic skin, is in adult skin only transiently synthesized after wounding, though exclusively by dermal fibroblasts (also for general review: [14, 78, 94, 103, 149]). A wider laminin-111 distribution in embryonic tissues suggested in earlier reports was due to the erroneously assigned specificity of a monoclonal antibody (4C7) to the laminin *α*1 instead of the *α*5 chain [22, 150, 151].

## 3. Identification of Molecular BM Defects by Genetic Approaches

Since defects in the structural components of BM, that is, respective laminins and collagen IV, are not compatible with early embryonic development, we like to focus here mainly on deletions or functional defaults of the bridging molecules nidogen and perlecan. Complete perlecan deficiency is lethal for mouse embryos at the midgestational stage primarily due to heart failure [104, 105]. Besides anomalies in cartilage and bone formation, particularly vascular BMs were seriously affected which presumably explains the extensive internal bleeding for vessel leakiness. Generally, perlecan is very important for developmental angiogenesis, so the deficiency of that could be largely responsible for organ failure in these embryos [34, 116].

Genetic ablation of nidogen-1 [152] or nidogen-2 [153] alone did not cause obvious BM alterations. However, in nidogen-1 null mice, a redistribution and increase of nidogen-2 are observed, for example, in skeletal and heart muscle or nerves suggesting that generally nidogen-2 can compensate the loss of nidogen-1 for BM formation [152, 154]. Nevertheless, nidogen-1 null mice show certain developmental and neurological defects indicating only partial redundancy [155–157]. Mice lacking both nidogens die perinatally from lung and heart anomalies, directly related to BM defects, while in most other tissues including the dermoepidermal junction in skin BMs they appear largely unaffected [17, 106]. The crucial role of nidogens in organ development was also confirmed in embryonic tissue models *in vitro* [113, 145]. At birth the skin of nidogen-null mice fulfills regular barrier functions revealing no obvious water loss (inside out) and complete resistance against dye penetration (outside in). However, examining skin ultrastructure some abnormal-looking basal cells were observed as well as microblistering and leakiness of small vessels (detailed later on). Interestingly, mice with deletion of the nidogen binding site on the laminin *γ*1 chain showed specifically defects of the urogenital tract, kidney, and lung but not any detectable anomalies of the cutaneous and capillary BMs [158]. Presumably that is due to compensation by nidogen-2 which is in contrast to nidogen-1 retained within the BM, implying alternative binding sites [115], while in addition the presence of laminins with a *γ*3 chain, harboring respective binding sites, may play a role as well. 

Another, apparently restrictive, regulator of BM assembly “extracellular matrix protein 1”/ECM1 came across when analyzing skin biopsies of lipoid proteinosis (LP) patients with mutations in the ECM1 gene (see [89, 90, 159]). The most striking clinical symptoms are hoarseness of the voice and mild, but progressive, mental retardation. Biopsies of scaling skin lesions revealed multiple BM duplications [159] and severe microvascular aberrations, marked by huge concentric BM deposits around small vessels, frequently leading to luminal collapse ([117]; see below). 

## 4. Incomplete Epidermal Reconstruction by Isolated Human Cells in Conventional Culture

Epidermal keratinocytes and dermal fibroblasts, representing the two main cell types of skin, have been analyzed extensively by cell culture *in vitro* for studies on skin physiology, repair, and tumorigenesis. However, major drawbacks of those approaches are that (i) both cell types behave very differently in conventional cultures on plastic dishes and (ii) in addition they intensively communicate with each other or the ECM *in vivo* which regulates growth and gene expression determining the skin phenotype. In the dermis fibroblasts are embedded in ECM (collagen, fibronectin, and proteoglycans) and acquire a spindle-shaped morphology [11], being only connected over long cell extensions via gap junctions [48], which differs completely from their flattened shape *in vitro*. Differently, keratinocytes form coherent cell layers *in vitro* like *in vivo*, undergoing epidermal differentiation. However, this process is incomplete and resembles somehow a regenerating wound epithelium. To a great part this is due to the conventional (two-dimensional) culture conditions, where the direction of nutrient supply is reversed from basal cell attachment sites *in vivo *(at the BM, facing dermis) to the upper epithelial surface (*in vivo* providing the water barrier). Despite of the different physiology of human and adult mouse skin, these changes were comparable in keratinocyte cultures from newborn mouse or human tissue [160–162]. An important achievement for the development of human cell models was the establishment of the human epidermal cell line HaCaT [163]. Like normal keratinocytes HaCaT cells respond to the induction of differentiation processes, for example, by growing cultures to very high cell densities, raising Ca^2+^, or lowering retinoid levels [5, 164, 165]. On the contrary, benign and malignant HaCaT-ras variants (containing a mutated Ha *ras*-gene) generally maintained their more complex, atypical keratin profiles *in vitro*, by and large related to their tumorigenic properties (see below; [164–166]).

## 5. Human Cells Rebuild Epidermal Architecture in Mouse Xenografts

Transplants of cultured mouse keratinocytes on the back of immune compatible mice had demonstrated the full differentiation potential of these cells, exposing them again to an authentic microenvironment [160, 161]. Likewise keratinocytes from human skin, hair follicle/outer root sheath cells, or HaCaT cells transplanted on nude mice ([163, 167–170]; also for further references) were able to restore epidermal tissue showing regular differentiation. Keratin patterns were normalized; that is, basal cells expressed keratin K5/14 and cells in the layers above keratin K1/10, followed by late differentiation markers, responsible for epidermal barrier function. The full restoration of epidermal architecture matched with the formation of a regular BM and mature hemidesmosomes [169, 170]. Monitored by immunofluorescence, the BM components were laid down sequentially, laminin-332 appearing first, shortly followed by nidogen, and with some delay by a *γ*1 chain laminin, presumably laminin-511, and collagen IV. Accordingly integrins, initially decorating cell surfaces in lower layers, became restricted in distribution, that is, *β*1 integrins to the basal cell surface and *α*6*β*4 mainly to the cell-matrix interface. Furthermore cell proliferation decreased as visualized by BrdU uptake, finally labelling about 5% of basal cells, a rate seen in normal epidermis [170] which is compatible with a role of BM in epidermal growth regulation. The gradual formation of the BM zone and epidermal-BM anchoring structures was confirmed at the ultrastructural level by EM, which underlines, together with immunostaining, that certain threshold levels of BM constituents are required for complete assembly. 

In order to study tumor-related defects of epidermal anchorage (BM, hemidesmosomes), tissue polarity, and differentiation in an experimental human tumor model benign and malignant HaCaT-ras cells were transplanted on nude mice, revealing unbalanced, but non-invasive or invasive, tissue-destructive growth, respectively [64, 171]. Reflecting mutual interactions with the changing stromal support, even malignant HaCaT-ras cells formed initially well polarized, differentiating epithelia with some remnant BM structures. However, this was changing dramatically with the mounting tumor-stroma reaction, showing in close correlation to the malignant properties the persistence of inflammatory cell infiltration and angiogenesis [35, 172], commonly downregulated in benign cell grafts or late wounds. Consequently, epithelial polarity declined showing irregular clusters of proliferating and differentiating cells. As hallmark, seen in skin squamous cell carcinomas [173], nonepidermal “simple” keratins K8/K18 and vimentin (an indicator for epithelial-mesenchymal transition/EMT) appeared at the invading front [171]. The areas of proliferation, expanding suprabasally, were strongly decorated by *β*1-integrins and *α*6*β*4 [64], similar to changes observed in mouse models of two-stage carcinogenesis ([63]; references in [174]). In the malignant cell grafts regular BM structures were completely disappearing, whereas laminin-332 increased aberrantly lining also lateral cell surfaces and deep epithelial clefts which was preceeding invasive growth. Nevertheless BM components and anchoring structures were still detectable by immune EM though they were displaced and diffusely distributed [175]. Comparable changes in laminin-332 or *γ*2 chain expression and location have been observed in squamous cell carcinomas of human skin and other carcinomas ([176, 177]; for review [65]).

## 6. Organotypic (3D) Cocultures Forming Artificial Skin *In Vitro*

In contrast to conventional culture models with their fundamental limitations regarding the relevance for skin physiology or pathology, the tissues generated by cell grafts on mouse are very complex, depending also largely on systemic effects and inflammatory host responses. To provide a better defined, simpler experimental system which still mimics the basic criteria of skin physiology, organotypic cocultures were established based on essential elements of skin [146, 178–183]. In this three-dimensional (3D) coculture system keratinocytes grow on collagen I matrices populated with dermal fibroblasts, using filter inserts and multiwell culture devices. Epithelial polarity is achieved by the media supply from underneath and epithelial surface exposure to air, that is, the incubator gas phase. While simulating the constellation in skin or keratinocyte transplants, the 3D model allows supplementation with diffusible molecules or factors, providing a controlled, closed system. Furthermore, genetically manipulated mouse or human cells, including presumptive progenitor or stem cells, can be combined which has been demonstrated for cells with deleted, silenced, or inducible gene expression [32, 103, 118, 149, 184–187]. With several combinations of normal cells from different sources, including human hair follicle, a regular epidermal phenotype could be reconstituted expressing respective differentiation markers [168, 183, 188] and normal BM structures [89, 180, 181, 183, 189–191]. This setup provides also an attractive alternative to test functions of mutated or gene manipulated cells of patients with BM/junctional defects for applications in gene therapy [141, 192, 193].

## 7. Distinct Functions of Perlecan and Nidogen Reconstructing a Dermoepidermal Interface

As stated already, perlecan is made by both cell types, while nidogen-1 and -2 originate from fibroblasts and the BM-associated laminin-332 and -511 (in skin) from keratinocytes [144, 146, 149, 191]. In the 3D model ablation of “dermal” perlecan had no deleterious effect on BM deposition or epithelial morphology, when using fibroblasts from perlecan-null embryos [105] with normal HaCaT cells [32]. Also the combination of perlecan deficient HaCaT cells (expressing perlecan antisense RNA) with wild-type fibroblasts (producing perlecan) had no effect on BM deposition as judged by light microscopy. However, a markedly delayed onset of epithelial growth was observed, while regular epidermal structures developed eventually [32]. This indicates that at least in this model perlecan has no effect on initiating BM assembly though firm perlecan incorporation in skin BM has been reported very recently [84]. Besides this apparently more stabilizing function, perlecan is certainly indispensable for functional BM properties such as control of balanced growth and other signaling cues [33, 116, 194]. Interestingly, total perlecan deficiency in the 3D model did not interfere with proliferation but dramatically enhanced the apoptosis of the epithelial cells [32]. Nidogen seemed to have a diametrically opposed effect in this regard. Whereas epithelial growth and differentiation remained virtually normal in the complete absence of nidogen or when interfering with nidogen interactions, this was devastating for deposition and assembly of BMs in the 3D model as outlined in detail below.

## 8. Nidogen Plays an Essential Role for BM Assembly *In Vitro*

For bridging of laminin and collagen IV networks nidogen-laminin binding had been assumed to be the initial step [99, 101, 108, 109]. Apart from those reports, this was also concluded from early appearance of nidogen together with laminin-111 in development ahead of a visible BM [41], BM assembly on live cells [14, 81], and the early deposition of nidogen at the dermoepidermal interface seen in cell grafts [169, 170]. Thus, as first functional proof in 3D coculture we interfered with this interaction by employing a laminin *γ*1 fragment (*γ*1III3-5 module) harboring the binding site in the *γ*1III4 module [112, 195]. Repeated application abolished the deposition of nidogen as well as laminin and perlecan at the matrix interface when examined by indirect immunofluorescence. Other components, such as laminin-332, collagen IV, and integrin *α*6*β*4 were only moderately affected, showing still a distinct continuous staining at the interface. BM assembly could be reverted by delayed onset or reactivated by the discontinuation of the treatment, respectively, demonstrating the dynamics of this process. So, already assembled BM structures disappeared again by late treatment with the *γ*1 fragment, while BM formation was resumed when treatment was halted. Epidermal morphology and differentiation remained largely normal as judged by staining for K1/K10 and “late” markers. Examining ultrastructure revealed that the *γ*1 fragment completely blocked BM formation (no *lamina densa* visible) and further abolished the formation of hemidesmosomal adhesion complexes. Consequently keratin filaments retracted from the ventral cellular aspect, while basal cells adhered directly to “dermal” collagen fibrils. 

Remarkably, immune EM revealed that BM constituents and hemidesmosomes were still present, though somewhat reduced and widely dispersed. This was in line with analyzing protein extracts of separated dermal and epidermal parts of cocultures. Thus, the major BM components nidogen-1, collagen IV, laminin-511 (laminin-10), and laminin-332 were detected by immunoblotting at similar levels with no signs of aberrant processing ([189]; compare nidogen-null below). Collectively the major defects observed in this setting were the lack of BM and epidermal adhesion structures, basal dissociation of the keratin network, and direct basal cell contacts to type I collagen fibrils. 

In order to demonstrate alternatively a direct role of nidogen itself, fibroblasts from knockout mice lacking either one or both nidogens were employed in 3D cocultures [149]. Like the blockage of binding [189], absence of both nidogens totally impaired BM deposition and structural assembly, while the amounts of all other BM components remained unchanged as shown by immunoblotting. This in addition confirmed that also under those conditions no measurable compensatory nidogen synthesis occurred in the keratinocytes. Similarly, immune EM revealed scattered distribution of BM components over a broader area, escaping detection by immunofluorescence. Furthermore, a dosage effect was observed using fibroblasts from heterozygous or homozygous nidogen-deficient mice which synthesize different, reduced nidogen levels. The functional potential of the two nidogens could be ultimately proven by supplementing nidogen-depleted 3D cocultures with either recombinant nidogen-1 or -2. Both restored the BM zone seen by immunofluorescence or EM showing a regular ultrastructure, underlining the functional redundancy between both nidogens for the assembly processes [149]. However, more recent studies, applying distinct binding domains of either nidogen-1 or -2 for BM reconstitution, provide clear evidence for distinct binding activities which may participate in tissue- or organ-specific effects not readily apparent by former molecular binding studies [103]. 

The striking differences between dermoepidermal BM formation *in situ* and in 3D cocultures indicate that tissue-related molecular modifications or “minor” components may play a role in addition to chemical and mechanical properties of the dermal ECM. Still another factor, not favoring BM assembly in this 3D model, is the low collagen I concentration in comparison to dermis and the relatively large volume of culture medium, both allowing fairly free diffusion of BM molecules. This is limiting the critical concentrations required for polymerization and assembly of BM structures, an effect recently termed “molecular crowding” [196]. Actually this might enhance the effects of nidogens in 3D cocultures, apparently catalyzing or stabilizing initial molecular interactions for BM formation which should be also more crucial for BM repair or remodeling *in vivo* [149]. Nevertheless, it has to be stated that BMs can *per se* develop *in vivo* in the absence of nidogens, however, with some restrictions and not in all organs suggesting a tissue-specific requirement for nidogens. According to recently reported immune EM studies, in skin laminin and collagen IV network were more intensely linked by perlecan aggregates than by nidogens which may reflect a progressed state of BM maturation [84]. This could also mean that the supramolecular structures of collagen IV and laminin-511 are more divergent than commonly assumed, revealing a general decrease of laminin-511 or -521 in BM of adult versus juvenile skin [197], higher levels being restricted to the space below hemidesmosomes [13]. In addition, there is clear evidence for other than structural BM functions (i.e., as crosslinker or adapter) of nidogen which apparently involves signaling events though both may be interlinked. Just giving a few examples: nidogen has been reported to rescue mammary epithelial cells from apoptosis [198] and, bound to laminin-111, to enhance laminin-driven gene expression and differentiation in the mammary gland [199]. In the epidermal 3D coculture model nidogen deposition closely correlates with the restriction of epidermal cell growth [18], and on the other hand it accelerates epidermal wound repair [200]. It is tempting to speculate that nidogen exerts that effect by acting as adapter or changing conformation of laminin, which could also apply to the revival of epidermal growth potential or stemness by laminin-511/-521 [66, 197] which may relate to protective effects on embryonic stem cells [67]. Of particular interest is also that nidogen-1 specifically participates in nerve path-finding while its ablation is causing seizures ([17, 115, 152, 155], also for review). Further, nidogen has been localized in neuromuscular junctions in *C. elegans*, together with collagen XVIII playing an important role for structural organization [28]. At least some of these effects should be mediated by binding of nidogens or such complexes to integrins like *α*3*β*1 or *α*v*β*3 [201–203] or to other cell surface receptors ([29, 44, 46], for general review [111]).

## 9. Cutaneous Microvasculature Is More Severely Affected by Molecular BM Defects

Macroscopically and histologically, the skin of mice lacking both nidogens appears to be fairly normal, showing no anomalies of the dermoepidermal BM by immunofluorescence [106]. Only some abnormal looking basal cells, rare microblisters, and slightly smaller hemidesmosomes (on average) have been observed by EM (E18.5; [17]). Because pups are dying around birth, one could only speculate what the later fate of epidermis might be during the transition from newborn to adult mouse skin with a dense hair coat and a much thinner epidermis. Unfortunately, the generation of mice with skin-specific nidogen deletion for further follow-up studies seems to be rather problematic and is currently not available (RN, data on reproduction of transgenic mice). In contrast to their intact cutaneous BM, the nidogen-deficient fetuses or newborns revealed mild intradermal bleedings indicating some microvascular defects. According to immune staining, in small vessels a defined BM was largely missing and instead an irregular, patchy pattern was observed with marked reduction of collagen IV, perlecan, and particularly of laminin-411 (laminin-8). As seen by ultrastructure, small blood vessels had thin leaky walls, completely lacking a distinct BM, showing dissociation of perivascular cells/presumptive pericytes and extravasation of erythrocytes [17]. This closely resembles the small leaky vessels around experimentally induced human skin tumors of squamous cell carcinoma type in nude mice [172, 175]. Nidogen destabilization or turnover may also be involved in vessel sprouting. This was markedly enhanced when we injected beads with adsorbed laminin *γ*1III3-5 fragments (blocking nidogen binding) next to those experimental tumors in nude mice (DB, unpublished data).

Collectively the data indicate that in skin primarily the laminin composition of the two BM types (Figure 2(e)) dictates if nidogens are required or not for BM assembly or stabilization. Laminin-511 (with three short arms for self-assembly) is generally absent in tip cells of sprouting vessels [80, 204–206] and also, according to our data, in small vessels of mice directly after birth, containing at that time point mainly laminin-411 which is unable to form networks by self-polymerization (Figure 2(e); [207]). Contrarily, larger vessels normally having both laminins display a regular BM also in absence of nidogens. Of note, additional, “associated” collagen types like collagen XV and XVIII [208] seem to regulate the thickness of the collagen IV mat and thus of BMs. Interestingly, proteolytic fragments of all three collagen types or perlecan restrict angiogenesis especially in cancer [209–216], which may concurrently normalize the tumor microvasculature and reduce vessel leakiness as observed previously when interfering with experimental tumor angiogenesis [172]. Interestingly in this context, laminin-332 has also been detected in tumor vessels and stromal cells like myofibroblasts, thus providing guiding tracks for migrating tumor cells in metastasis or anchorage for cell arrest, respectively, at vessel walls and distant secondary sites [37, 38, 40, 217].

A reverse picture was seen in skin biopsies of lipoid proteinosis (LP) patients. In this disorder dysfunction or lack of ECM1 causes excessive BM deposition at the dermoepidermal junction but most pronounced around small vessels, where these enormously sized, multiple BMs obviously impair vascular function ([117, 159]; also for review, [89, 90]). Ultrastructurally, the epidermal adhesion structures/anchoring fibrils below hemidesmosomes (composed of collagen VII; schematic view in Figure 2(a)) were markedly displaced though components like laminin-332 and collagen VII remained partially associated [117]. Concerning functional consequences, a crucial role of both collagen VII and processed laminin-332 (including the C-terminal *α*3 G45 fragment) has been proposed for invasiveness of carcinomas in several reports, which rather show an extensive turnover of other BM components [65, 218, 219]. Albeit this issue is still controversial, since patients with recessive dystrophic epidermolysis bullosa, lacking regular collagen VII expression can still develop invasive skin tumors [220]. 

## 10. Summary and Further Outlook 

For skin barrier function the regular structural organization of the epidermis is an unequivocal requirement which depends on an intact BM as anchor and support. The current state of the art allows no simple answer for ultimate molecular mechanisms to build up a fully functional BM. Being classically placed in the center of BM assembly the two nidogen isoforms revealed in the *in vitro* skin model that they can both induce and accelerate BM formation including epidermal BM adhesion structures. Furthermore the data strongly suggest that nidogens also function as instant stabilizers of molecular interactions, which is particularly important for fast tissue or BM remodeling. Perlecan, on the other hand, apparently provides more spacious links between laminin and collagen IV networks (“spot-wedded,” [84]) which may be also beneficial for BM texture and mechanical properties under steady-state conditions. An interesting question would be, if this spacing corresponds to the increased laminin-511 deposition beneath hemidesmosomes, demonstrated previously [13]. So it would be further of great interest if the nidogen concentration closely follows this proposed spotty laminin-511 arrangement.

The combination of genetic and 3D coculture approaches in future studies appears promising to define further isoform-specific effects, as reported for laminin-511/521 [66] and molecular interactions playing a role in skin physiology, formation of appendages, and skin pathology [54, 197, 221, 222]. Other players like ECM1 [90] or “minor” collagens like type V [87], XV, and XVIII [31, 88] shall be considered as well, as they may serve as organizers or nucleus for BM assembly or implement BM microheterogeneity. The latter could be of particular relevance for the postulated epidermal stem cell niche [68, 223–227]. Thus, there is substantial evidence for direct influences of ECM or BM properties on stem cell fate or behavior [66, 69–72] which basically applies for other tissue-specific, precursor, or mesenchymal stem cells [228, 229] and embryonic stem cells as well [230]. In this respect a crucial step forward has been recent improvements of the “dermal” part of this 3D model, approaching physiological and mechanical features of an authentic dermis [188, 231], while BM production in particular may be further enhanced by specific supplements [232]. To explain the discrepancies between the *in vitro* and *in vivo* models, a promising task would be the generation of mouse strains with skin-specific constitutive or inducible ablation of both nidogens. Though not an easy task, this would permit to study BM formation or stability and epidermal barrier function in adult animals avoiding the deleterious systemic drawbacks. Last not least it is of great medical interest that compromised BM structures in tissues and the vascular system are a major hallmark of cancer progression and invasiveness. Nidogens exhibit a rather high susceptibility against proteases like matrix metalloproteases [195, 233], meprins [234, 235], or cathepsin S [236] getting highly activated in tumors. As such nidogens could serve at the front line as targets for early attacks leading to the destruction of tissue barriers and vascular leakiness facilitating tumor cell spreading and metastasis.
